# Cell-Free Metabolic Engineering: Recent Developments and Future Prospects

**DOI:** 10.3390/mps2020033

**Published:** 2019-04-30

**Authors:** Hye Jin Lim, Dong-Myung Kim

**Affiliations:** Department of Chemical Engineering and Applied Chemistry, Chungnam National University, Daejeon 34134, Korea; yhjysy@naver.com

**Keywords:** cell-free metabolic engineering, cell-free protein synthesis, bioconversion platform

## Abstract

Due to the ongoing crises of fossil fuel depletion, climate change, and environmental pollution, microbial processes are increasingly considered as a potential alternative for cleaner and more efficient production of the diverse chemicals required for modern civilization. However, many issues, including low efficiency of raw material conversion and unintended release of genetically modified microorganisms into the environment, have limited the use of bioprocesses that rely on recombinant microorganisms. Cell-free metabolic engineering is emerging as a new approach that overcomes the limitations of existing cell-based systems. Instead of relying on metabolic processes carried out by living cells, cell-free metabolic engineering harnesses the metabolic activities of cell lysates in vitro. Such approaches offer several potential benefits, including operational simplicity, high conversion yield and productivity, and prevention of environmental release of microorganisms. In this article, we review the recent progress in this field and discuss the prospects of this technique as a next-generation bioconversion platform for the chemical industry.

## 1. Introduction

Owing to recent advances in genetic and genomic engineering techniques, microbial cells are increasingly being used as self-replicating microreactors that can produce diverse materials from exogenously introduced genes [1,2]. However, the use of living cells often prevents us from harnessing their full synthetic power. Living systems operate only within narrow condition ranges, including temperature, salt concentration and solvent properties. Toxicity or metabolic burden also limit high-volume production of recombinant products. In addition, the interconnectedness of cellular metabolic pathways often reduces substrate flux into synthetic pathways, thus lowering product yield and conversion efficiencies. Most of these problems stem from the requirement of living cells to maintain balanced homeostasis [3]. Liebig’s law of the minimum teaches us that deterioration of any essential cellular component can result in failure of the entire system, thus preventing the operation of the desired pathways. 

In theory, many of these problems can be avoided by using the individual biological components specifically required to produce the target products. In fact, the use of purified biosynthetic machinery in cell-free systems has a long history that spans several decades. A prominent example is the use of purified recombinant DNA polymerase. Purified DNA polymerase can be used for many more tasks than it performs in living cells. In addition to its common use for rapid DNA amplification in thermal cyclers, the DNA synthesis activity of DNA polymerases has been widely used for many applications in combination with various reagents and conditions, including diagnostic techniques and genetic mutagenesis [4]. 

Cell-free use of biosynthetic machinery has also been expanded to protein production, which is more complicated and requires many enzymes and translational factors. These components were purified or extracted from cells and successfully reconstituted to produce recombinant proteins directed by genetic programming contained in the reaction mixtures [5,6,7,8,9]. Cell-free metabolic engineering is the latest addition to these recent efforts to harness cellular functions outside of cells and it involves the use of purified or crude enzymes to produce chemical compounds [10,11]. Liberated from the requirement of maintaining cellular viability and growth, cell-free metabolic engineering provides far greater design flexibility and wider operational conditions for synthetic metabolic pathways. Cell-free metabolic engineering systems also offer important benefits that cannot be attained using living cells, including quantitative and precise assessment of performance by direct sampling, rapid cycles of design-build-test iterations and the capability to use non-natural or non-biological components. While the concept of cell-free metabolism was introduced as early as 100 years ago with the demonstration of ethanol production in crude yeast lysate [12], the use of enzymes has long been relegated to an auxiliary role in the production of structurally complex intermediates via organic synthesis approaches. However, growing demand for cleaner and more efficient chemical processes along with notable advances in genetic engineering and enzyme technology have led to recognition of cell-free synthetic approaches as a promising method for synthesizing the diverse range of chemical compounds used in industrial implications.

This review summarizes recent efforts to harness the principle of cell-free synthesis to reproduce intracellular reaction pathways outside of a model system and to reach yields and productivity that are not achievable with current cell-based methods. In particular, our discussion focuses on two closely related topics: synthesis of enzymes that catalyze chemical conversion pathways with industrial implications and production of important chemicals via cell-free use of the necessary enzymes. We also discuss the potential to integrate cell-free enzyme synthesis and metabolic engineering to build DNA-programmed, cell-free metabolic engineering systems.

## 2. Cell-Free Protein Synthesis Systems

### 2.1. Development of Highly Productive Cell-Free Protein Synthesis Systems

**Figure 1:**
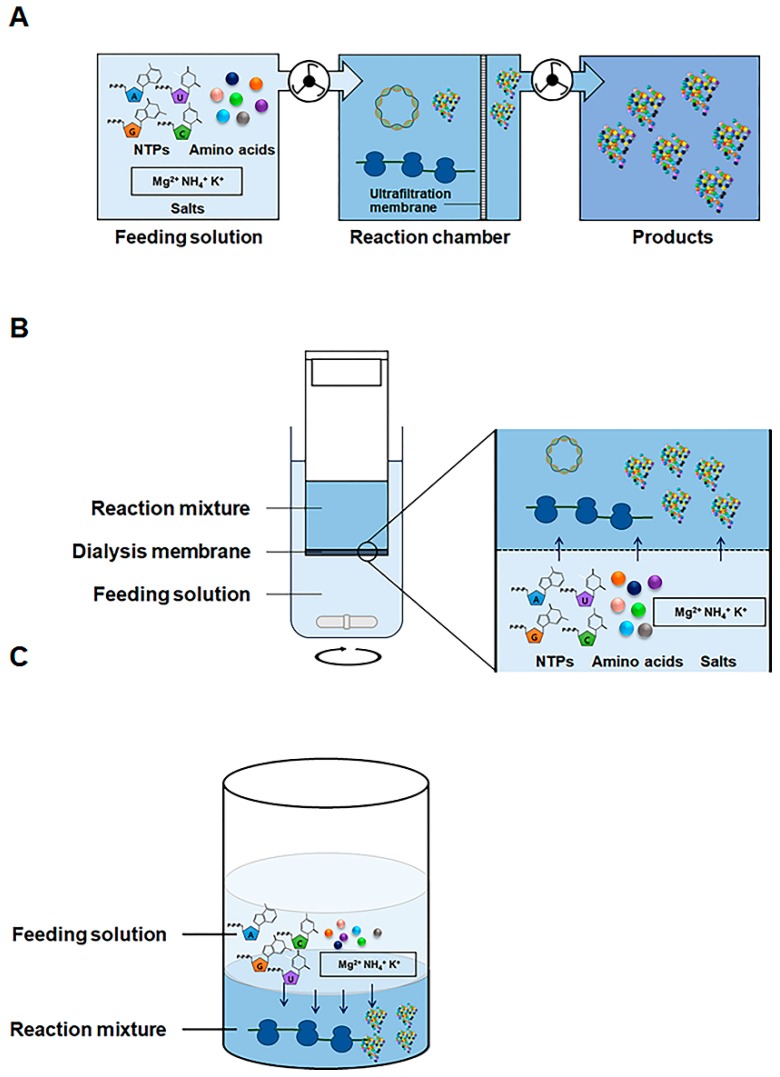
Reaction configurations of cell-free protein synthesis for continuous supply of substrates. (**A**) A continuous flow cell-free translation system. The feeding solution containing the substrates for protein synthesis continuously through the reaction mixture retained by an ultrafiltration membrane. (**B**) A continuous exchange cell-free protein synthesis system. The supply of substrates and removal of by-products are achieved by diffusional exchange through a dialysis membrane. (**C**) A bilayer cell-free protein synthesis system. Feeding solution is overlaid on top of the reaction mixture for cell-free protein synthesis and diffusional exchange of substrates and by-products take place at the interface of the two phases.

Similar to the case of PCR, the operational convenience and productivity of cell-free protein synthesis approaches have evolved over the last decades. For example, extensive studies on the factors limiting conventional cell-free protein synthesis systems have revealed that a steady and continuous ATP supply is one of the most important requirements for efficient protein production [13,14,15]. Each step of the molecular process of protein synthesis (aminoacylation, transcription, and translation) consumes large amounts of ATP. This consumption leads to a rapid decrease in the ATP level in the reaction mixture and limits the productivity of conventional cell-free protein synthesis systems [16,17]. Use of high concentrations of ATP or other energy sources cannot easily address this problem because of the resulting accumulation of inorganic phosphate, which inhibits protein synthesis by chelating magnesium ions, an essential cofactor required for this process [18,19]. Early attempts to address this dilemma involved continuous ATP supplementation and removal of inorganic phosphate via forced pumping [20,21] or diffusional exchange (Figure 1) [17,22,23].

**Figure 2:**
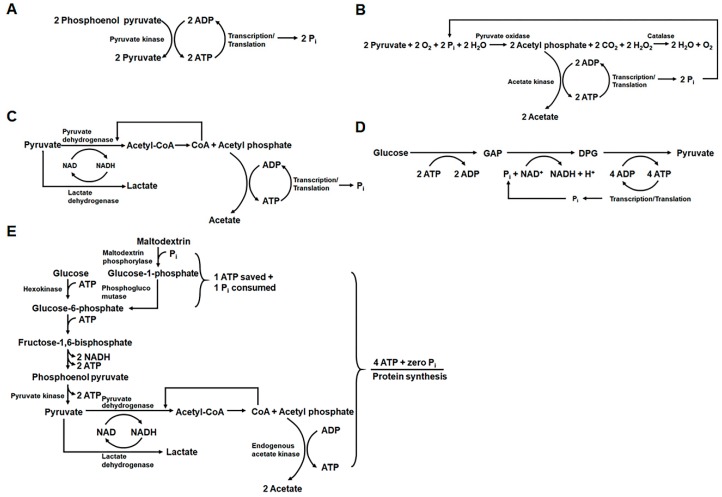
Biochemical strategies for enhanced supply of ATP without the accumulation of inorganic phosphate. (**A**) Conventional methods for ATP regeneration during cell-free protein synthesis simply rely on the substrate-level phosphorylation of ADP using the energy sources with high-energy phosphate bonds (i.e., phosphoenolpyruvate, creatine phosphate, and acetyl phosphate). In this scheme, inorganic phosphate accumulates in the reaction mixture in amounts proportional to those of the energy sources. (**B**) Kim and Swartz demonstrated that pyruvate can be used as a phosphate-free energy source, in combination with exogenously added pyruvate oxidase [16]. In their scheme, pyruvate and recycled inorganic phosphate produce acetyl phosphate, which is subsequently used to regenerate ATP. (**C**) Generation of acetyl phosphate from pyruvate can also be achieved by using the endogenous enzymes in the cell extract. (**D**) Use of glucose to regenerate ATP via glycolytic pathway in the cell extract. (**E**) Use of maltodextrin as a secondary energy source. ADP: adenosine diphosphate; ATP: adenosine triphosphate; Pi: inorganic phosphate; NAD: nicotinamide adenine dinucleotide; NADH: reduced nicotinamide adenine dinucleotide; GAP: glyceraldehyde-3-phosphate; DPG: 1,3-diphosphoglycerate.

While these approaches markedly improved the duration of the reaction and thus the final target protein yield, they had the drawback of requiring complex devices and excessive amounts of reagents [24]. Therefore, different strategies have been developed to improve the ATP supply during batch cell-free protein synthesis reactions, while avoiding inorganic phosphate accumulation (Figure 2). In 1999, Kim and Swartz developed a method for sustained ATP supplementation in a batch cell-free protein synthesis system without inorganic phosphate accumulation. Instead of phosphate-containing energy sources, they used pyruvate as a phosphate-free energy source to regenerate ATP [16]. Because pyruvate is the final product of the glycolytic pathway, their results inspired the use of glucose and glycolytic intermediates as energy sources for regenerating the ATP required for protein synthesis [25,26].

In subsequent studies, the ATP regeneration efficiency was further improved by using polymeric glucose as a high-density energy source [27,28]. Most recently, Caschera and Noireaux demonstrated that polyphosphate can be used as a highly efficient and cost-effective energy source for high-yield protein production in a cell-free protein synthesis system [29]. As a result of these efforts, production of milligram quantities of recombinant proteins in batch reactions is now routinely reported.

### 2.2. Direct Programming of Cell-Free Protein Synthesis with Linear DNA Templates

**Figure 3:**
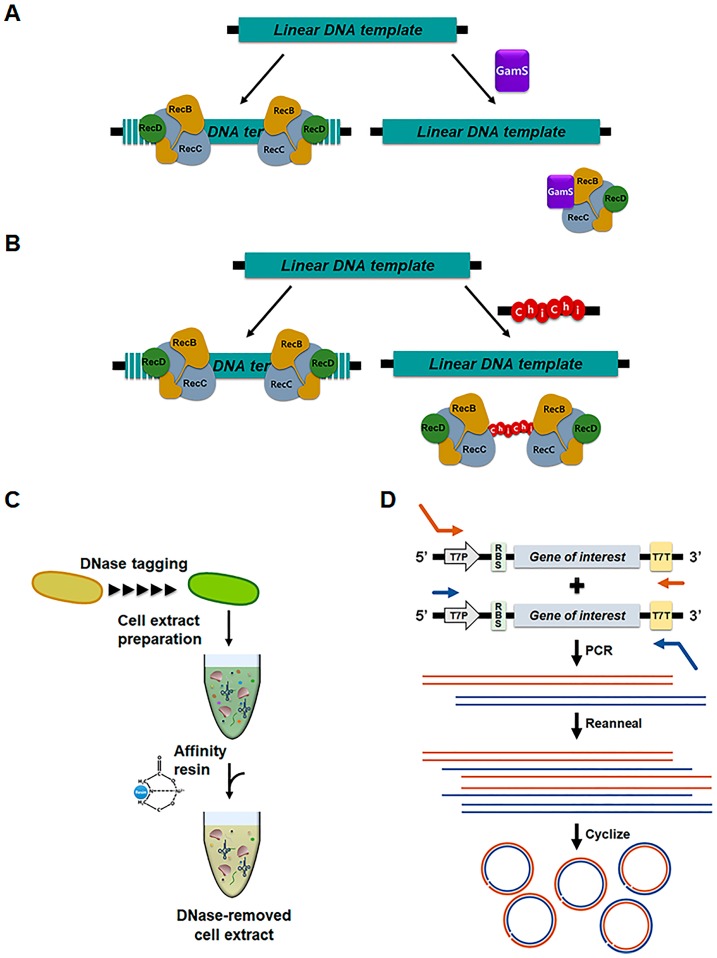
Strategies for improving the stability of linear DNA templates in cell-free protein synthesis systems. (**A**) Stabilization of linear template by use of GamS protein, an inhibitor of RecBCD complex. (**B**) Sequestration of RecBCD complex by using a short DNA containing repeated χ-site. (**C**) Affinity-removal of DNases from the cell extract. (**D**) Cyclization of linear templates by endogenous DNA ligase in the cell extract.

Another important issue in cell-free protein synthesis is the method used to prepare the template DNA. When cell-free protein synthesis is directed by plasmid-borne genes (like in cell-based expression systems), it is still necessary to grow cells for cloning and template DNA amplification. This requirement off-sets the benefits of cell-free protein synthesis; however, the need for cell growth can be avoided by using PCR to prepare the template DNA. In general, the efficiency of cell-free synthesis directed by PCR-amplified DNA is substantially lower than that in reactions containing plasmid-borne templates. This effect is mainly due to rapid degradation of the linear templates by exonucleases present in the cell-free extract [30]. Numerous attempts have been made to address the issue of template DNA stability during cell-free protein synthesis (Figure 3). Sitaraman et al. developed a method to stabilize PCR-amplified linear DNA using the lambda phage Gam protein, which inhibits the RecBCD exonuclease [31]. Marshall et al. introduced Chi-sites into the template DNA to block RecBCD without the need for purified Gam protein [32]. Seki et al. improved the efficiency of cell-free protein synthesis from linear DNA templates via affinity-removal of polynucleotide phosphorylase (PHPase) and RecD from the cell extract [33]. Wu et al. designed stable linear templates cyclized between single-stranded 5′-phosphorylated overhangs by the endogenous ligase activity of *Escherichia coli* S30 extracts [34]. Ahn et al. took the alternative approach of stabilizing the mRNA transcribed from the linear DNA, rather than stabilizing the DNA itself. They found that the mRNA lifespan is remarkably extended when its 3′-end forms a stem-loop structure and the cell-free protein synthesis is conducted in an extract lacking RNase E activity. This enhanced mRNA stability, in turn, led to highly efficient protein expression at yields comparable to those obtained from reactions using plasmid templates [35]. By eliminating the time-consuming steps required for template preparation, these methods enable direct programming of cell-free protein synthesis systems for the instant production of the desired proteins.

### 2.3. Cell-Free Enzyme Synthesis

**Figure 4:**
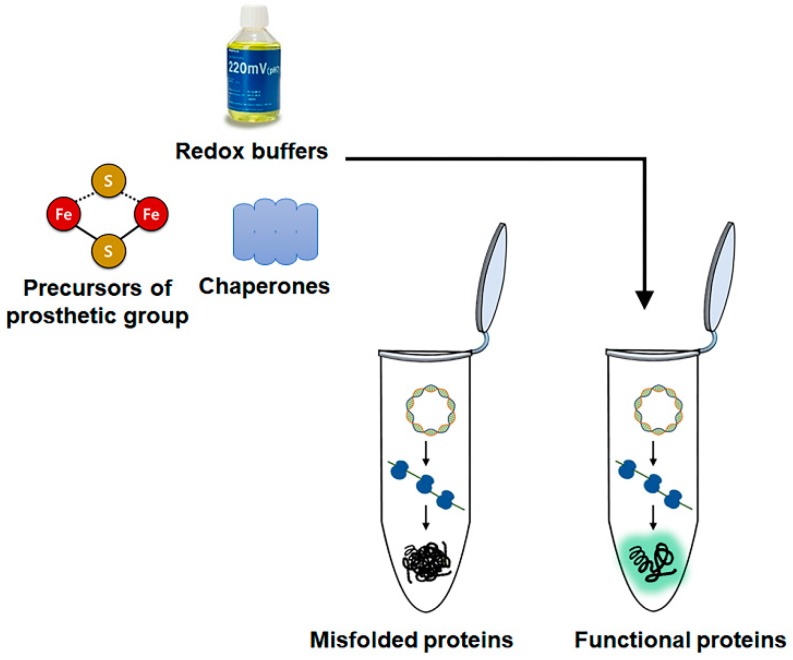
Enhanced production of functional enzymes by direct additions of folding effectors. The open nature of cell-free protein synthesis allows direct additions of various chemicals and biomolecules that assist proper folding of desired enzymes.

As described above, cell-free protein synthesis techniques have rapidly evolved to produce large amounts of recombinant proteins directly from in vitro-produced template DNA. These advances have been successfully combined with the versatile nature of cell-free protein synthesis to produce enzymes that are otherwise difficult to express in functional forms [36] (Figure 4).

For example, functional *Candida antarctica* lipase B can be produced by simply adjusting the redox potential of the cell-free protein synthesis reaction mixture to facilitate intramolecular disulfide bond formation [37,38]. Cell-free synthesis also allows facile introduction of unnatural amino acids into an enzyme structure, which is particularly useful for producing enzymes that can be immobilized in controlled orientations. For example, through site-specific introduction of an unnatural amino acid containing a chemical handle, Wu et al. could immobilize T4 lysozyme on solid beads. It was found that optimal orientation of immobilization allowed substantially enhanced activity and stability of the immobilized enzyme [39]. Swartz et al. demonstrated the versatility of cell-free protein synthesis systems for producing complex enzymes by expressing functional [FeFe] hydrogenase. They could produce and mature algal and bacterial hydrogenases using *E. coli* extracts containing the HydG, HydE, and HydF proteins. Pre-incubation of these proteins with sulfide and iron in the reaction mixture allowed proper assembly of the iron-sulfur cluster and apoenzyme during the subsequent hydrogenase synthesis [40]. In a similar approach, Li et al. successfully expressed functional multicopper oxidase, which has potential biotechnological applications. This enzyme commonly shows low expression levels in traditional recombinant hosts; however, by simply adding copper sulfate to the reaction, the cell-free protein synthesis system yielded over 1 mg mL^−1^ of soluble and functional multicopper oxidase [41]. Kwon et al. used P450 BM3 as a proof-of-concept model to show that the pathways for prosthetic group and apoenzyme synthesis could be combined in a one-pot reaction to produce functional monooxygenase [42]. Cell-free protein synthesis systems are also a promising platform for producing enzymes that are toxic to recombinant hosts. For example, Lim et al. reported successful phospholipase A1 production using a cell-free protein synthesis system derived from E. coli. Phospholipase A1 degrades phospholipids in the cell membrane and, thus, cannot be efficiently produced in the cytoplasm of live *E. coli* cells. By decoupling enzyme expression from cell physiology in a cell-free protein synthesis system, they achieved an over 1000-fold higher yield of functional phospholipase A1 [43]. 

## 3. Cell-Free Metabolic Engineering

### 3.1. Purified Protein-Based Cell-Free Metabolic Engineering

The conditions for bioconversion using living cells are restricted by the physiological limits required to maintain life. In most cases, for example, microbial processes can only be operated below 40 °C to prevent cellular damage [44]. Furthermore, metabolic pathways that involve intermediates or products that are toxic to the host cells cannot be easily used. The complexity of cellular metabolism is another barrier for efficient target compound production. Due to these features, most natural microbes are not efficient enough to support high-yield production of target chemicals sufficient to meet the demands of current petroleum-based markets [45]. The most straightforward solution to these limitations is to use purified enzymes to build cell-free metabolic pathways. Cell-free metabolic pathways based on purified enzymes enable simple interpretation of results and optimization of the participating enzymes. For example, Bujara et al. successfully demonstrated how cell-free metabolic pathways can be optimized by coupling them to real-time analysis methods [46]. Cell-free enzymatic approaches allow flexible design of novel pathways solely focused on target molecule production. In an effort to eliminate the ATP-driven reactions required for the conversion of glucose into pyruvate, Guterl et al. developed an artificial glycolytic pathway that requires only four enzymes. Their simplified pyruvate synthesis pathway was subsequently streamlined via addition of enzymatic pathways for ethanol and isobutanol synthesis [47]. Despite the advantage of being free from the constraints imposed by cells, a shortcoming of cell-free metabolic pathways is that they are disconnected from the cellular biochemical replenishing systems. Korman et al. introduced a modular design for an artificial 27-enzyme pathway for cell-free monoterpene production. Through smart design and arrangement of the modules of the enzymatic pathways that produce intermediates and regenerate co-factors, such as ATP and NADPH, they built a balanced cell-free monoterpene synthesis process with a conversion yield of greater than 95% and titers greater than 15 g L^−1^ [10].

These results clearly demonstrate the potential of cell-free metabolic engineering for the production of industrial chemicals via artificial pathways. In addition to chemical production, Martin et al. developed a synthetic pathway that produces 10 moles of dihydrogen via consumption of one mole of ATP during xylose breakdown [48]. Furthermore, cell-free enzymatic pathways have also been successfully used for bioelectricity production. Zhu et al. reported a synthetic cell-free pathway that produces nearly 24 electrons per glucose unit in an aerobic enzymatic fuel cell. This enzymatic fuel cell exhibited an energy-storage density one order of magnitude higher than that of lithium-ion batteries [49].

### 3.2. Cell Extract-Based Cell-Free Metabolic Engineering

**Figure 5:**
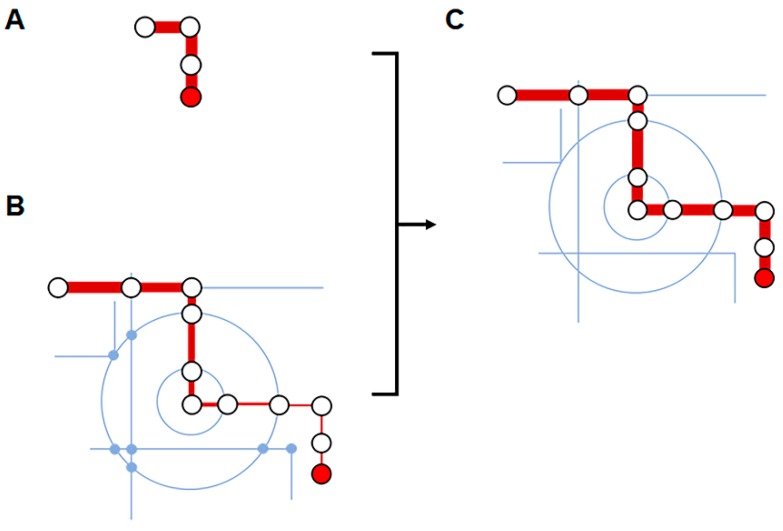
Cell-free metabolic engineering based on cell extract. (**A**) Enzyme-based cell-free metabolic engineering offers greater flexibility and controllability. However, the requirement for purified individual enzyme limits its use for multi-step bioconversion. (**B**) On the other hand, cell-based metabolic engineering suffers from the complexity of cellular metabolic pathways and the fluxes of intermediates can be reduced by their cellular consumptions (represented by gradual reduction of the thickness of red line). (**C**) Cell-free metabolic engineering can hitchhike the cellular metabolism, while minimizing genetic modification of host cells and loss of intermediates.

Despite the attractive advantages of enzyme-based cell-free pathways, the requirement for laborious purification of individual enzymes limits their use, particularly for multistep reactions. In cases where the intermediates of the final products can be generated via cellular metabolism, a meet-in-the-middle strategy employing cell lysates might be a more realistic approach [50,51,52,53]. Such an approach would harness the activities of the cellular components after removal of the membrane barrier and complement the cellular pathway that converts a raw material into the necessary intermediates by introducing additional enzymes required to generate the final product (Figure 5). For example, Bujara et al. produced a series of unnatural monosaccharides from glucose by adding enzymes to the E. coli lysate that complete the final synthesis steps. The cell-free metabolism intrinsic to the E. coli lysate resulted in accumulation of dihydroxyacetone phosphate (DHAP), which was subsequently converted into unnatural monosaccharides via the actions of exogenously added enzymes [54]. In a similar approach, Kay and Jewett established a cell-free metabolic pathway for 2,3-butanediol production. In their study, the pyruvate synthesized by the cell-free glycolytic pathway was then successfully converted to 2,3-butanediol via acetolactate and acetoin through exogenous addition of the required enzymes (acetolactate synthase, acetolactate decarboxylase and butanediol dehydrogenase) to the reaction mixture [11]. The titer of the cell-free-synthesized 2,3-butanediol reached 80 g L^−1^, which is close to the theoretical yield. Yi et al. proposed an interesting alternative approach for 2,3-butanediol synthesis based on a hybrid cell-free synthesis system. The cyanobacterial endogenous starch-breakdown pathway was combined with the E. coli glycolytic pathway by mixing lysates of the two species. As this mixed-lysate system accumulated pyruvate from starch, addition of acetolactate synthase, acetolactate decarboxylase and butanediol dehydrogenase led to successful 2,3-butanediol synthesis. These results demonstrate that the synthesis of new heterologous metabolic pathways could support biomolecule synthesis [55].

## 4. Conclusions

Cell-free metabolic engineering is expected to provide an alternative route for biological production of chemical compounds. As systems developed through cell-free metabolic engineering are independent of cell viability and growth and insulated from toxicity of the synthesized chemicals, they can offer increased flexibility and higher conversion efficiency. While most of the studies on this topic, including those discussed in this review, have been conducted using purified or extracted enzymes, an interesting approach would be to integrate cell-free metabolic engineering with PCR and cell-free protein synthesis to establish a directly programmable metabolic engineering platform [56]. For this concept to be developed into a practical method, additional techniques will be needed, including methods for expressing functional proteins and for regulating the expression levels of exogenous enzymes. Considering the marked progress in the development of such techniques [57,58,59], it is likely that genetically programmed and controlled cell-free metabolic engineering platforms will soon emerge.
